# Mitotic p21^Cip1/CDKN1A^ is regulated by cyclin-dependent kinase 1 phosphorylation

**DOI:** 10.18632/oncotarget.10330

**Published:** 2016-06-30

**Authors:** Nina-Naomi Kreis, Alexandra Friemel, Brigitte Zimmer, Susanne Roth, Michael A. Rieger, Udo Rolle, Frank Louwen, Juping Yuan

**Affiliations:** ^1^ Department of Gynecology and Obstetrics, J. W. Goethe-University, D-60590 Frankfurt, Germany; ^2^ Department of Hematology/Oncology, J. W. Goethe-University, D-60590 Frankfurt, Germany; ^3^ German Cancer Consortium (DKTK), Heidelberg, Germany; ^4^ German Cancer Research Center (DKFZ), Heidelberg, Germany; ^5^ Department of Pediatric Surgery and Pediatric Urology, School of Medicine, J. W. Goethe-University, D-60590 Frankfurt, Germany

**Keywords:** p21 phosphorylation, mitosis, Cdk1/cyclin B1, p21 stability, chromosome segregation

## Abstract

The multifunctional protein p21^Cip1/CDKN1A^ (p21) is an important and universal Cdk-interacting protein. Recently, we have reported that p21 is involved in the regulation of the mitotic kinase Cdk1/cyclin B1 and critical for successful mitosis and cytokinesis. In the present work we show that S130 of p21 is phosphorylated by Cdk1/cyclin B1 during mitosis, which reduces p21′s stability and binding affinity to Cdk1/cyclin B1. Interfering with this phosphorylation results in extended mitotic duration and defective chromosome segregation, indicating that this regulation ensures proper mitotic progression. Given that p53, the major transcriptional activator of p21, is the most frequently mutated gene in human cancer and that deregulated Cdk1 associates with the development of different types of cancer, this work provides new insight into the understanding of how deregulated p21 contributes to chromosomal instability and oncogenesis.

## INTRODUCTION

Cyclin-dependent kinases (Cdk), a family of proline-directed serine/threonine kinases, regulate the cell cycle [1]. Cdk1 is essential for the mitotic entry and its inactivation is a prerequisite for the mitotic exit [2, 3]. The Cdk inhibitor p21^Cip1/CDKN1A^ (p21) is an intrinsically unstructured protein [4], which binds to both Cdk and/or cyclin subunits [5–7]. It is involved in cell cycle regulation, DNA repair, apoptosis, cell motility, gene transcription, stem cell reprogramming, and senescence induction [5, 8]. p21 is often reduced in human cancer, partially through loss of functional tumor suppressors such as p53 or hyperactive oncogenes like c-myc. Its expression is induced upon diverse cellular stress stimuli and it loses its tumor suppressor function when it localizes to the cytoplasm [5, 8]. The degradation of p21 is a complex and fine-tuned process depending on the cell cycle stage, binding partners, its localization and post-modifications [9]. It has been recently reported that acetylation counteracts ubiquitination and stabilizes p21 [10], whereas methylation facilitates phosphorylation [11]. Phosphorylation alters p21′s activity, localization, binding partners and stability [5, 12]. In fact, p21 contains 14% potential phosphorylatable amino acid residues [5]. It is phosphorylated by various Cdks like Cdk2/cyclin A at T57 [13], Cdk2/cyclin E [14] and Cdk6/cyclin K at S130 [15]. Moreover, p21 is phosphorylated at Y77/Y151 *in vitro* by non-receptor tyrosine kinases, which influences its Cdk/cyclin binding and stability [16].

We have recently shown that p21 is involved in the regulation of the mitotic progression, whereby its loss prolongs mitotic duration resulting in defects in chromosome segregation and cytokinesis [6]. Moreover, loss of p21 increases the efficiency of Polo-like kinase 1 (Plk1) inhibitors [17]. During those studies, we observed that p21 is abundantly present in mitosis accompanied by slowly migrating bands in different tumor cell lines, in particular, when cells face stress [6], suggesting that p21 is post-modified in mitosis, possibly phosphorylated. We wondered the responsible kinase and the relevance for the mitotic process. In the present work, we have systematically addressed these issues.

## RESULTS

### p21 is phosphorylated during early mitotic stages

**Figure 1:**
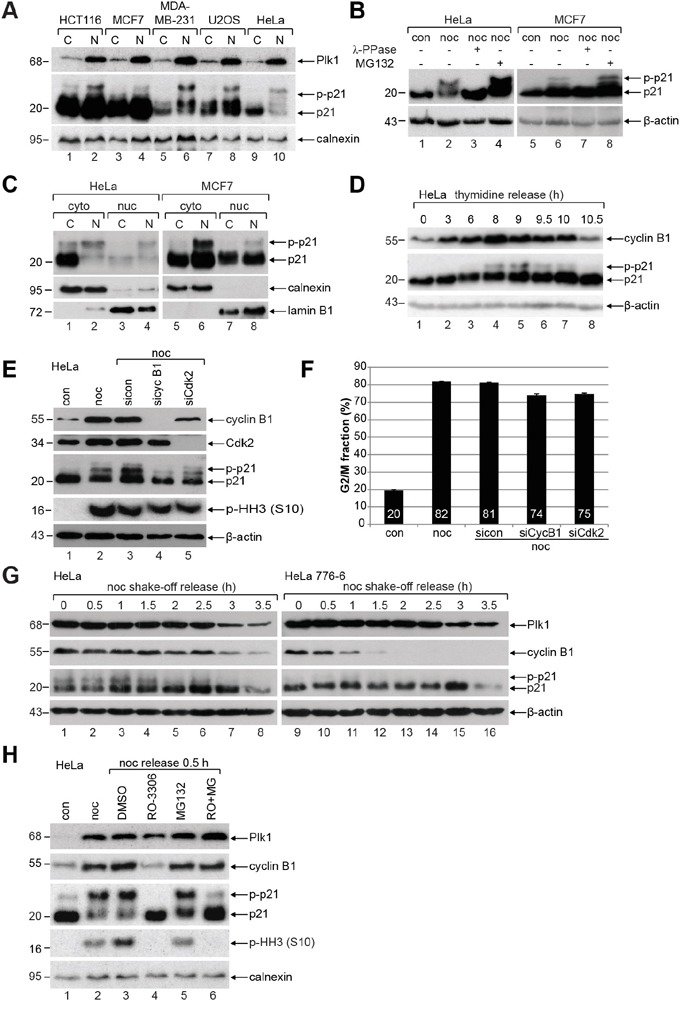
p21 is phosphorylated during mitosis in diverse cancer cell lines **A.** HCT116, MCF7, MDA-MB-231, U2OS and HeLa cells were untreated (C) or synchronized to prometaphase with nocodazole (N) for Western blot analyses with indicated antibodies (p-p21: phosphorylated p21). Calnexin served as loading control. **B.** HeLa and MCF7 cells were untreated (con), treated with nocodazole (noc) or nocodazole combined with λ-phosphatase (λ-PPase) for Western blot analysis. Nocodazole treated cells were further incubated with the proteasome inhibitor MG132 as positive control. β-actin served as Western blot loading control. **C.** Cytoplasmic (cyto) and nuclear extracts (nuc) were prepared from untreated (C) or nocodazole (N) treated HeLa and MCF7 cells. Calnexin and lamin B1 served as cytoplasmic and nuclear marker, respectively. **D.** HeLa cells were synchronized with a double thymidine block and released in fresh medium for indicated time points. β-actin served as loading control. **E.** HeLa cells transfected with control siRNA (sicon), siRNAs targeting cyclin B1 (sicyc B1) or Cdk2 (siCdk2) were synchronized to prometaphase with nocodazole (noc) for Western blot analysis. Untreated (con) and nocodazole treated cells (noc) without transfection served as controls. β-actin was the loading control. **F.** Cell cycle distribution was analyzed by FACS. The G2/M peak was quantified and results of the duplicates are shown as mean ± SD. **G.** HeLa cells or stable HeLa 776-6 cells expressing shRNA targeting human cyclin B1 were synchronized with nocodazole (noc). Shake-off cells were released into fresh medium and harvested at indicated time points for Western blot analyses with indicated antibodies. β-actin served as loading control. **H.** HeLa cells were synchronized to prometaphase with nocodazole and released for 0.5 h into fresh medium containing DMSO, 6 μM RO-3306 (RO), 10 μM MG-132 (MG) or both inhibitors. Untreated (con) and nocodazole (noc) treated cells served as controls. Calnexin was the loading control.

To corroborate the existence of slowly migrating bands of mitotic p21, colon carcinoma HCT116, breast cancer MCF7 and MDA-MB-231, osteosarcoma U2OS and cervical carcinoma HeLa cells were untreated or synchronized to prometaphase for Western blot analysis (Figure 1A). In prometaphase, when the mitotic marker Polo-like kinase 1 (Plk1) is highly expressed (Figure 1A, 1^st^ panel, lanes 2, 4, 6, 8 and 10), p21 is clearly present accompanied by an appearance of several additional slowly migrating bands indicative of being post-modified (Figure 1A, 2^nd^ panel, lanes 2, 4, 6, 8 and 10). To address if these slowly migrating bands are induced by phosphorylation events, we treated cells additionally with λ-phosphatase (λ-PPase) and the band/bands disappeared (Figure 1B, 1^st^ panel, lanes 3 and 7), suggesting that the slowly migrating band/bands of p21 are indeed induced by phosphorylation events. Moreover, when cells were treated with the proteasome inhibitor MG132, p21 was stabilized in prometaphase, both fast and slowly migrating bands (Figure 1B, 1^st^ panel, lanes 4 and 8). Cytoplasmic-nuclear separation of HeLa and MCF7 cells revealed that phosphorylated p21 is more abundant in the cytoplasm than in the nucleus of prometaphase cells (Figure 1C, 1^st^ panel, lanes 2 and 6). To follow the p21 phosphorylation status over the cell cycle, thymidine release kinetics was performed. When the cyclin B1 level peaked at 8 h post release, p21 was phosphorylated, which disappeared with the degradation of cyclin B1 (Figure 1D), supporting a mitotic phosphorylation event.

To define the mitotic kinase responsible for this phosphorylation, HeLa cells were treated with siRNA targeting cyclin B1, the regulatory subunit of Cdk1, or siRNA against Cdk2, and synchronized to prometaphase for Western blot analysis. Compared to control siRNA treated cells (Figure 1E, 3^rd^ panel, lane 3), phosphorylated p21 almost disappeared in cells deficient of cyclin B1 (Figure 1E, 3^rd^ panel, lane 4), whereas phosphorylated p21 receded only slightly in cells treated with siRNA targeting Cdk2 (Figure 1E, 3^rd^ panel, lane 5), indicating that Cdk1 could be the main kinase for phosphorylating mitotic p21. The signals of phospho-histone H3 (p-HH3) were slightly reduced yet still abundant in cells knocked down of cyclin B1 or Cdk2, indicating that most of these cells with left cyclin B1 or Cdk2 were still able to enter mitosis (Figure 1E, 4^th^ panel). This was further supported by cell cycle analysis showing a reduction of 6-7% in the G2/M population in cyclin B1 or Cdk2 downregulated cells relative to control siRNA treated cells (Figure 1F). To reinforce these results, HeLa or HeLa 776-6 cells, which stably express shRNA against cyclin B1 to reduce its level [18, 19], were synchronized to prometaphase. Mitotic cells were obtained by shake-off and released into fresh medium for indicated time points for Western blot analysis. Relative to control HeLa cells, HeLa 776-6 cells displayed almost no phosphorylated p21 (Figure 1G, 3^rd^ panel), further underscoring that phosphorylated p21 is associated with the activity of Cdk1. Moreover, the phosphorylation signal of p21 was abolished in mitotic HeLa cells released into medium containing RO-3306, a specific and efficient Cdk1 inhibitor [20, 21], whereby the amount of the fast migrating p21 was increased (Figure 1H, 3^rd^ panel, lane 4). To halt RO-3306 treated cells in mitosis, mitotic cells were treated additionally with the proteasome inhibitor MG132. In this case, the phosphorylation signal of p21 was definitely decreased (Figure 1H, 3^rd^ panel, lane 6), whereas the levels of the mitotic markers Plk1 and cyclin B1 were hardly changed (Figure 1H, 1^st^ and 2^nd^ panel, lane 6), suggesting that the reduction of the phosphorylation signal is ascribed to the reduced Cdk1 kinase activity. Of note, RO-3306 treated mitotic cells showed hardly the p-HH3 (S10) signal (Figure 1H, 4^th^ panel, lanes 4 and 6), which is in line with the previous observation [21], indicative of the importance of Cdk1 for mitosis. In sum, these data strongly point to the notion that the phosphorylation of p21 is mainly linked to the activity of Cdk1 in mitosis.

### p21 is phosphorylated by Cdk1/cyclin B1 at S130

**Figure 2:**
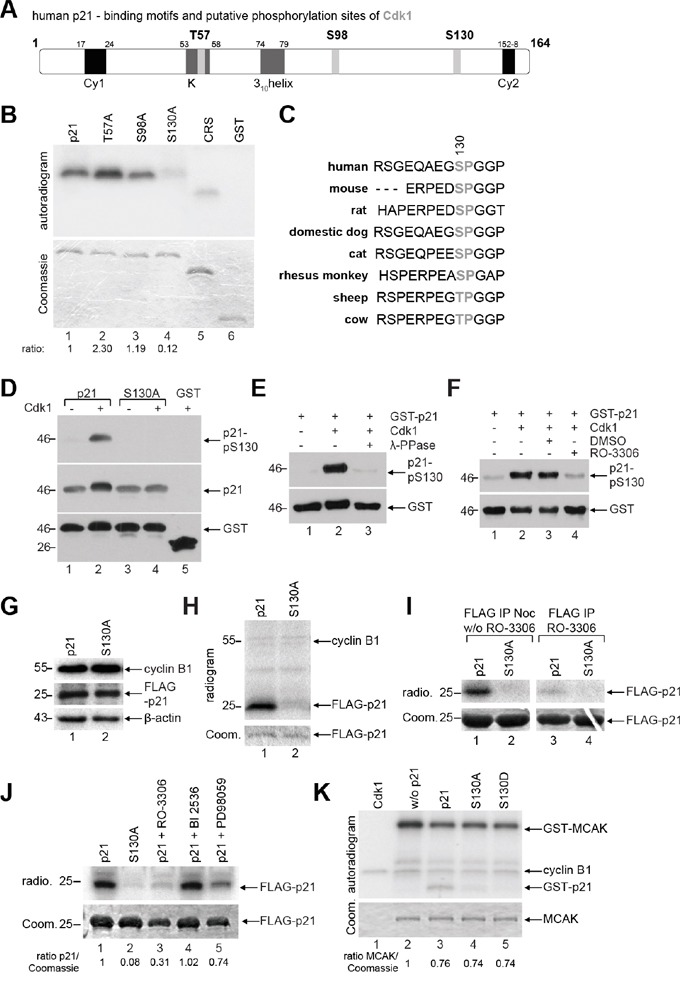
p21 is phosphorylated at S130 by Cdk1/cyclin B1 **A.** Schematic illustration of human p21 including its putative phosphorylation sites of Cdk1 with the minimal consensus motif pT/pS-P (light gray). Its two cyclin binding motifs Cy1 and Cy2 (black), the Cdk binding site K and the 3_10_helix (dark gray) are also shown. **B.** *In vitro* kinase assay of human GST-tagged p21 and its mutants, whereby each threonine or serine was replaced by alanine. CRS (cytoplasm retention signal) of cyclin B1 and GST proteins were taken as positive and negative control, respectively. The same gel was stained with Coomassie as input control. The phosphorylation intensities, relative to the input, are shown, evaluated by using ImageJ (National Institutes of Health). **C.** Sequence alignment of p21 from different species and the S130 residue is highlighted in gray. **D-F.** Different *in vitro* studies were carried out with the generated phospho-antibody against p21-pS130. (D) GST-p21 and its mutant S130A were subjected to *in vitro* kinase assay with non-radioactive ATP and Cdk1 kinase and further analyzed by Western blot analysis with indicated antibodies. The same membrane was stained with GST antibody as loading control. (E) *In vitro* kinase assay was performed with GST-p21 as described in (D), in the presence of phosphatase (λ-PPase) and analyzed by Western blot analysis. The same membrane was stained with GST antibody as loading control. (F) *In vitro* kinase assay with GST-p21, in the presence of the Cdk1 inhibitor RO-3306, was performed, and analyzed by Western blot analysis. The same membrane was stained with GST antibody as loading control. **G.** HeLa cells transfected with FLAG-tagged p21 or its mutant S130A were synchronized with nocodazole (noc) for Western blot analyses with antibodies against cyclin B1 and FLAG tag as cell cycle and transfection efficiency control, respectively. β-actin served as loading control. **H.** *Ex vivo* kinase assay. The extracts from (G) were immunoprecipitated with FLAG^®^ M2 beads for a kinase assay without further addition of Cdk1/cyclin B1. The results are presented by an autoradiogram (upper panel). The Coomassie (Coom.) staining served as loading control (lower panel). **I.** Same experimental setup as in (H), incubation time without (w/o) and with addition of the Cdk1 inhibitor RO-3306 (9 μM). The results are presented by an autoradiogram (radio., upper panel). The Coomassie (Coom.) staining served as loading control (lower panel). **J.** *Ex vivo* kinase assay by using different kinase inhibitors: Cdk1 inhibitor RO-3306 (9 μM), Plk1 inhibitor BI2536 (25 nM) and MAP kinase cascade inhibitor PD98059 (10 μM). Ratio of p21 normalized against the Coomassie loading control is indicated. **K.** *In vitro* kinase assay of Cdk1/cyclin B1 with a known substrate MCAK (the mitotic centromere-associated kinesin), in the presence of GST-tagged wild type p21 or its mutants. The Coomassie (Coom.) staining of MCAK served as loading control.

The putative phosphorylation site of the mitotic master kinase Cdk1 with the minimal consensus sequence S/T-P [22, 23] is found three times in the human p21, namely T57, S98 and S130 (Figure 2A, highlighted in light gray). To map down the phosphorylation site, we replaced each of the potential sites with alanine in the GST-p21 construct utilizing site-directed mutagenesis. *In vitro* kinase assays were carried out with recombinant wild type GST-p21 and its mutants. While the phosphorylation signals of GST-p21T57A and GST-p21S98A were not reduced, the alanine substitution of S130 almost abolished the signal (Figure 2B, lane 4). Interestingly, S130 is highly conserved throughout mammals (Figure 2C), implying a conserved role in various species. To further verify that p21 is phosphorylated by Cdk1, a specific phospho-antibody targeting S130 in p21 was generated and used in various *in vitro* studies (Figure 2D-2F). The phosphorylation of S130 by Cdk1 was recognized by this phospho-antibody and the alanine substitution abrogated completely the signal (Figure 2D). Upon treatment with phosphatase, the phosphorylation signal was nearly disappeared (Figure 2E). Moreover, the phosphorylation signal was strongly decreased in the presence of the Cdk1 inhibitor RO-3306 (Figure 2F). These results suggest that the phospho-antibody works specifically *in vitro* and S130 in p21 is the major phosphorylation site for Cdk1. Unexpectedly, this antibody worked with a low specificity in cells, probably due to p21′s intrinsically unstructured nature and its “folding-on-binding mechanism” [7]. Instead, we performed a kinase assay *ex vivo*. HeLa cells were transfected with wild type FLAG-p21 or its non-phosphorylatable mutant FLAG-p21S130A and synchronized to mitosis for immunoprecipitations with FLAG^®^ M2 antibody beads. The precipitates were further subjected to an *in vitro* kinase assay in the presence of ^32^P-labeled ATP without adding additional Cdk1 kinase. The amounts of the transfected constructs (Figure 2G, 2^nd^ panel), the levels of precipitated wild type FLAG-p21 and its mutant FLAG-p21S130A (Figure 2H, lower panel, Coomassie staining) as well as the levels of precipitated cyclin B1 (Figure 2H, indicated in upper panel) were comparable. While wild type FLAG-p21 was phosphorylated by precipitated Cdk1/cyclin B1 (Figure 2H, upper panel, radiogram, lane 1), FLAG-p21S130A displayed only a slight signal (Figure 2H, radiogram, lane 2). The *ex vivo* experiments were further carried out by adding the Cdk1 inhibitor RO-3306 to the reaction. The phosphorylated wild type p21 was clearly reduced in the presence of the inhibitor (Figure 2I). In addition to the Cdk1 inhibitor, the *ex vivo* experiments were also performed in the presence of the Plk1 inhibitor BI 2536 as well as the MAP kinase cascade inhibitor PD98059 (Figure 2J), which is also known to phosphorylate p21 at S130 *in vitro* [24]. Among these kinase inhibitors, the Cdk1 inhibitor RO-3306 showed the strongest reduction of the phosphorylation signal of wild type FLAG-p21. Collectively, these results clearly indicate that Cdk1 is the major kinase, which phosphorylates p21 at S130 *in vitro* and *ex vivo*.

To reveal if the mutants affect the kinase activity of Cdk1, an *in vitro* kinase assay was performed with a known substrate of Cdk1/cyclin B1, the mitotic centromere-associated kinesin (MCAK) [22], in the presence of wild type GST-p21 or either of its mutants. Again, wild type GST-p21 was phosphorylated by Cdk1/cyclin B1 (Figure 2K, lane 3), but not its mutants. As p21 is not a proficient inhibitor of Cdk1/cyclin B1 *in vitro* [6, 25], a decline of about 25% in Cdk1/cyclin B1 kinase activity was observed in the presence of wild type GST-p21, which was comparable with either of the mutants (Figure 2K, lane 2-5, MCAK signal), implying that the phosphorylation status in p21 hardly affects the kinase activity of Cdk1.

### Phosphorylated p21 is less stable

**Figure 3:**
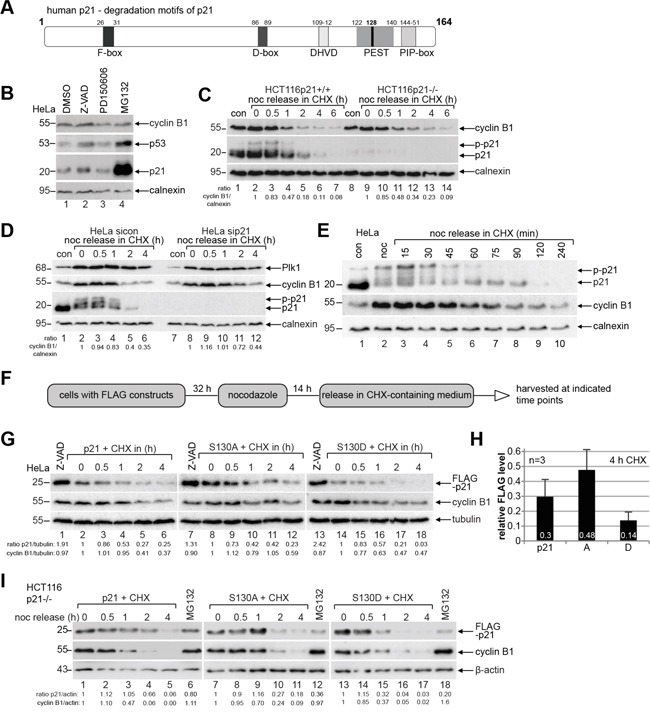
Endogenous phosphorylated p21 and p21S130D are less stable **A.** Schematic illustration of human p21 including its different known destruction motifs (gray boxes) like F-box, D-box, caspase cleavage site (DHVD), PEST sequence and PIP-box. **B.** HeLa cells were treated overnight with the pan-caspase inhibitor Z-VAD, the calpain inhibitor PD150606 and the proteasome inhibitor MG132 for Western blot analyses with indicated antibodies. DMSO served as vehicle and calnexin as loading control. **C.** HCT116 p21+/+ and p21−/− cells were synchronized to prometaphase with nocodazole (noc), released into CHX-containing medium for indicated time and harvested for Western blot analysis. Calnexin served as loading control. Ratio of cyclin B1/calnexin is shown. **D.** HeLa cells transfected with control siRNA (sicon) or siRNA against p21 (sip21) were synchronized to prometaphase with nocodazole (noc). The cells were released into CHX-containing medium. Calnexin served as loading control. Ratio of cyclin B1/calnexin is shown. **E.** HeLa cells were synchronized to prometaphase with nocodazole (noc), released into medium containing CHX for indicated time points for Western blot analysis. Non-treated or nocodazole treated cells were taken as controls. Calnexin served as loading control. **F.** Working schedule of the CHX kinetics. **G.** HeLa cells were transfected with different p21-constructs, synchronized with nocodazole and released in CHX-containing medium. Cells treated with the pan-caspase inhibitor Z-VAD were taken as control. Tubulin served as loading control. **H.** Quantification of the FLAG level normalized against the loading control is shown as mean ± SEM from three independent experiments. Time point: 4 h after CHX treatment. **I.** CHX release kinetics was also performed with HCT116 p21−/− cells expressing FLAG-p21, S130A or S130D. MG132 treated cells served as positive and β-actin as loading control.

The protein degradation and stability of p21 is well studied and it is known that the degradation is often triggered by its site-specific phosphorylation and different binding partners [5, 9, 26, 27]. p21 contains a plenty of degradation-related motifs: E3-ligase recognition motifs like F box [5] and D box [28], a PEST sequence [29] with a calpain cleavage site at S128 (belonging to the family of calcium-dependent, non-lysosomal cysteine proteases) [30], a PIP degron (substrates of CRL4 Cdt2) [31] and a caspase-3 cleavage site (DHVD) [32] (Figure 3A). As the proteasome-dependent pathways are well known for p21′s turnover [5, 9, 26, 27], we examined the E3-ligase-independent pathways like caspase and calpain cleavage. HeLa cells were treated overnight with the pan-caspase inhibitor Z-VAD or the calpain inhibitor PD150606 compared to vehicle control and the treatment with the proteasome inhibitor MG132, for Western blot analysis. Along with a strong accumulation of p21 upon treatment with MG132 (Figure 3B, 3^rd^ panel, lane 4), its protein stability was also affected by the pan-caspase inhibitor Z-VAD (Figure 3B, 3^rd^ panel, lane 2), whereas calpain cleavage inhibition impacted scarcely its protein turnover (Figure 3B, 3^rd^ panel, lane 3). Of note, p53 is increased after the inhibition of the caspase and the proteasome pathway, indicating that the accumulated p21 could result from both the stabilization of the protein and increased transcription via the p53 signaling. These data support the common observation that the degradation of p21 is majorly dependent on the proteasome pathway.

To study precisely the turnover of p21 during mitosis, HCT116 cells with and without p21 were synchronized to prometaphase and released into medium containing cycloheximide (CHX), a protein synthesis inhibitor, and harvested for Western blot analysis. p21 is a short-lived protein during mitosis (Figure 3C, 2^nd^ panel). Interestingly, compared to HCT116 cells with p21, cyclin B1 is more stable in HCT116 cells without p21 (Figure 3C, 1^st^ panel), which was also observed in HeLa cells treated with siRNA against p21 (Figure 3D, 2^nd^ panel), suggesting that the p21 status may influence directly the stability of cyclin B1 or indirectly via interfering with mitotic progression. Intriguingly, while non-phosphorylated p21 retained till 90 min in cells released from nocodazole treatment and incubated with fresh medium containing CHX (Figure 3E, 1^st^ panel, lane 8), the phosphorylated form was reduced at 45 min (Figure 3E, 1^st^ panel, lane 5) and undetectable at 75 min (Figure 3E, 1^st^ panel, lane 7), when cyclin B1, the regulatory subunit of Cdk1, started to be degraded (Figure 3E, 2^nd^ panel, lane 7). The data indicate that phosphorylation of p21 alters its turnover during mitosis.

To explore if the phosphorylation of p21 by Cdk1 impacts its stability, HeLa cells were transfected with wild type FLAG-p21 or its mutants, synchronized and released into CHX-containing medium (Figure 3F). Western blot analysis revealed that compared to wild type FLAG-p21 at 0 min (Figure 3G, 1^st^ panel, lane 2), non-phosphorylatable FLAG-p21S130A was more stable (Figure 3G, 1^st^ panel, lane 8), whereas phosphomimetic FLAG-p21S130D was less stable (Figure 3G, 1^st^ panel, lane 14). Moreover, FLAG-p21S130D degraded faster than wild type FLAG-p21 and FLAG-p21S130A during the release course and it was almost disappeared at 2 h in HeLa cells (Figure 3G, 1^st^ panel, lane 17). Further quantification analysis clearly indicates that FLAG-p21S130D is the most unstable form at 4 h (Figure 3H). Interestingly, FLAG-p21S130D was more affected by the overnight treatment with the pan-caspase inhibitor Z-VAD than wild type FLAG-p21 or FLAG-p21S130A (Figure 3G, 1^st^ panel, lane 1, 7 and 13) compared to nocodazole treated cells (Figure 3G, 1^st^ panel, lane 2, 8 and 14). To underscore these results, we transfected HCT116 p21−/− cells with wild type FLAG-p21 or its mutants and the CHX kinetics was carried out. In line with the observations from HeLa cells, FLAG-p21S130D was less stable compared to wild type FLAG-p21 and its non-phosphorylatable mutant FLAG-p21S130A (Figure 3I, 1^st^ panel). Notably, relative to wild type FLAG-p21 (Figure 3I, 1^st^ panel, lane 6), both mutants were hardly affected by the treatment with the proteasome inhibitor MG132 (Figure 3I, 1^st^ panel, lanes 12 and 18), suggesting strongly that S130 is involved in the proteasome dependent degradation of mitotic p21. Taken together, the data underline the notion that phosphorylation of S130 by Cdk1 renders p21 unstable.

### Non-phosphorylatable p21 binds strongly to Cdk1/cyclin B1

**Figure 4:**
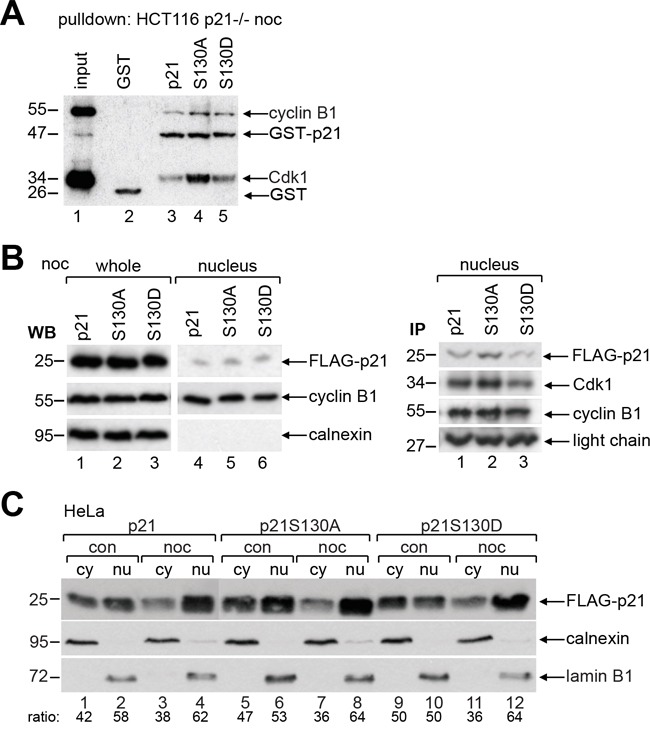
Non-phosphorylatable p21 binds strongly to Cdk1/cyclin B1 **A.** Pulldown assays were performed with nocodazole treated cellular extracts from HCT116 p21−/− cells in the presence of GST, GST-p21, GST-p21S130A or GST-p21S130D. **B.** HCT116 p21−/− cells were transfected with wild type FLAG-p21, FLAG-p21S130A or FLAG-p21S130D and synchronized to prometaphase with nocodazole. The amounts of transfected plasmids were adjusted, so that the expression levels of FLAG-p21 and its mutants were comparable. Left panel: whole cell extracts and nuclear extracts were prepared for Western blot analysis as transfection efficiency and input control for immunoprecipitation in the right panel. Calnexin served as cytoplasmic control. Right panel: immunoprecipitation of FLAG-transfected nuclear cell extracts with cyclin B1 antibody. Light chain served as loading control. **C.** HeLa cells transfected with wild type FLAG-p21 or its mutants were non-treated (con) or synchronized to prometaphase with nocodazole (noc) and cytoplasmic (cy) and nuclear extracts (nu) were prepared for Western blot analysis. Calnexin and lamin B1 served as cytoplasmic and nuclear extract marker, respectively.

To gain insight into the binding affinity of p21 and its mutants to Cdk1/cyclin B1, pulldown assays were performed using different GST-p21 recombinant proteins incubated with cellular extracts from mitotic HCT116 p21−/− cells. Compared to wild type p21 or the phospho-mimetic mutant p21S130D (Figure 4A, lane 3 and 5), p21S130A, the non-phosphorylatable mutant, bound strongly to Cdk1 and cyclin B1 (Figure 4A, lane 4). To substantiate this observation, HCT116 p21−/− cells transfected with wild type FLAG-p21 or its mutants were synchronized to prometaphase and nuclear extracts were prepared (Figure 4B) for immunoprecipitation with cyclin B1 antibody. Relative to wild type FLAG-p21 and FLAG-p21S130D (Figure 4B, right panel, lane 1 and 3), more FLAG-p21S130A was associated with Cdk1/cyclin B1 (Figure 4B, right panel, lane 2). These results reveal that non-phosphorylatable p21 binds Cdk1/cyclin B1 more tightly.

To analyze if phosphorylation changes p21′s subcellular localization, which has been reported for T145, S153 or T57/S130 [5, 24], HeLa cells transfected with wild type FLAG-p21, S130A or S130D were non-synchronized or synchronized to prometaphase, and cytosolic and nuclear extracts were prepared for Western blot analysis. Compared to wild type p21, the subcellular localization of both mutants was unchanged in the interphase as well as in mitosis (Figure 4C), indicating that the single phosphorylation of S130 barely impacts its location.

### Phosphorylation of p21 influences the mitotic progression

**Figure 5:**
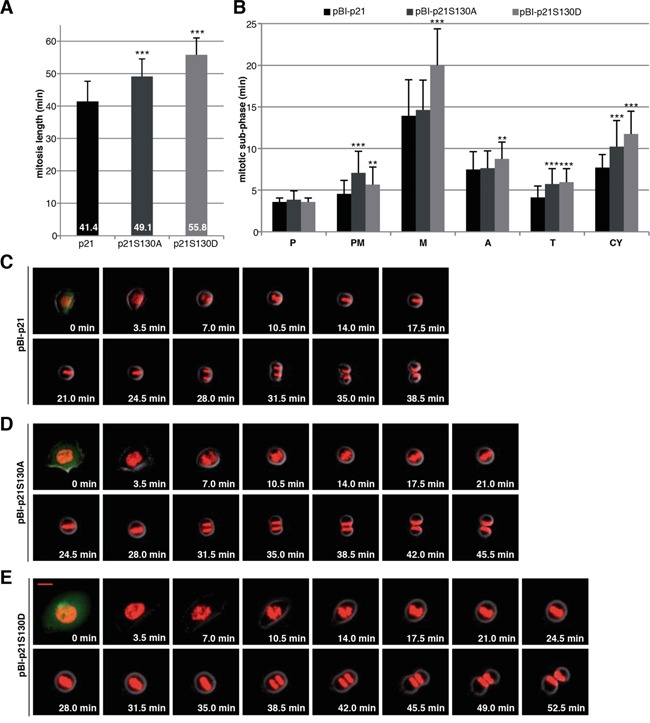
Interfering with phosphorylation of S130 in p21 extends mitotic duration **A.** HCT116 p21−/− cells stably marked with H2B-tdTomato and transfected with different pBI-p21 constructs were subjected to time-lapse imaging. pBI vector is a mammalian bidirectional expression vector designed to express a protein of interest and a green fluorescent protein (ZsGreen) as transfection marker shown at 0 min. The duration of mitosis, captured by time-lapse imaging at 3.5 min image intervals, was evaluated (n=50 cells of each condition). The results are presented as mean ± SD and statistically analyzed. ***p < 0.001. **B.** The time of each mitotic subphase was evaluated (n=50 cells per each cell line). The results are presented as mean ± SD and statistically analyzed between wild type pBI-p21 and pBI-p21S130A/D. **p < 0.01, ***p < 0.001. P: prophase, PM: prometaphase, M: metaphase, A: anaphase, T: telophase, CY: cytokinesis. **C-E.** Representative pictures of mitotic cells from pBI-p21 (C), pBI-p21S130A (D) and pBI-p21S130D (E) are shown. Scale bar: 20 μm.

To examine the effect of the phosphorylation at S130 on mitotic progression, time-lapse imaging was performed in living HCT116 p21−/− cells stably expressing fluorescent histones (H2B-tdTomato in red) [6], which were further transfected with wild type p21 or its mutants cloned in a pBI vector, which is a mammalian bidirectional expression plasmid designed to express a protein of interest and a green fluorescent protein served as a transfection marker. In our previous study, by tracking individual transfected mitotic cells, namely from chromosome condensation with round-up cell shape to the formation of the membrane of two daughter cells with decondensed chromosomes, we found that loss of p21 prolonged the duration of mitosis (44.5 min), relative to control HCT116 p21+/+ cells (37.1 min) [6]. We show here that the expression of wild type pBI-p21 was able to partially rescue the extended mitosis time of HCT116 p21−/− cells by showing a mitotic duration of 41.4 min, whereas cells expressing pBI-p21S130A needed 49.1 min and cells expressing pBI-p21S130D even 55.8 min to pass mitosis (Figure 5A). Further detailed analysis revealed that compared to cells transfected with wild type p21, cells with pBI-p21S130A had a prolonged prometaphase, whereas cells with pBI-p21S130D exhibited a remarkably extended metaphase (Figure 5B-5E), suggesting that phosphorylation of S130 is required for the proceeding into metaphase and subsequent dephosphorylation is desired for the initiation of anaphase. In addition, an extension in other subphases of mitosis was also observed in cells transfected with the different mutants (Figure 5B-5E), highlighting the importance of p21′s proper regulation throughout mitosis.

### p21 mutants are unable to rescue defective segregation in HCT116 p21−/− cells

**Figure 6:**
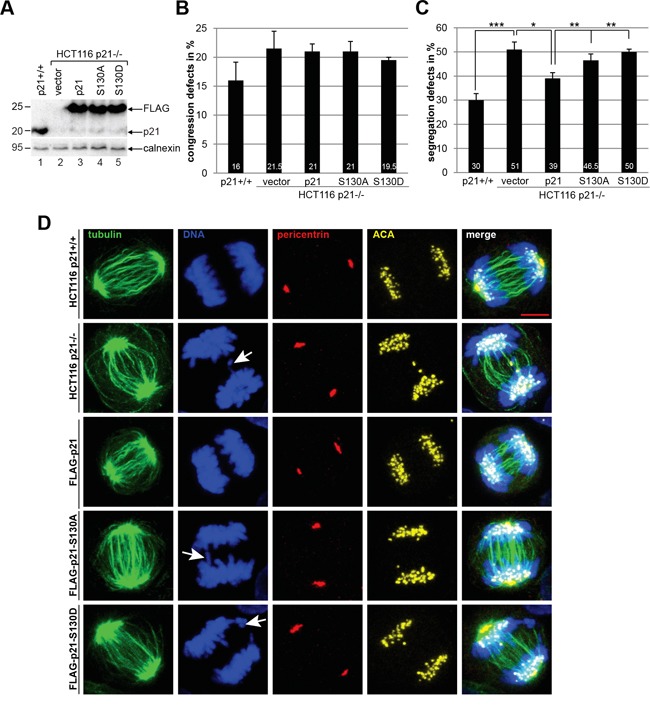
p21 mutants are unable to rescue defective chromosome segregation in HCT116 p21−/− cells Cells were transfected with FLAG vector, wild type FLAG-p21 or its mutants. The amounts of transfected plasmids were adjusted, so that the expression levels of FLAG-p21 and its mutants were comparable. HCT116 p21+/+ cells served as control. 48 h after transfection, cells were fixed and stained with antibodies as indicated and examined by confocal laser scanning microscopy. **A.** Western blot analysis was performed as transfection efficiency control. Calnexin served as loading control. **B.** Chromosomal congression defect was analyzed in metaphase cells (50 cells for each condition). The results are based on four independent experiments (200 cells for each condition) and presented as mean ± SEM. **C.** Chromosomal segregation failure was evaluated in anaphase cells (50 cells for each condition) and the results are derived from four independent experiments (200 cells for each condition) and presented as mean ± SEM. *p < 0.05, **p < 0.01, ***p < 0.001. **D.** Representative anaphase cells are shown. White arrows indicate defective chromosome segregation. Scale bar: 5 μm.

We were then interested in the mitotic phenotype of cells overexpressing wild type FLAG-p21 or its mutants. Transfected cells (Figure 6A) were stained for microtubule marker α-tubulin, centrosome marker pericentrin, the kinetochore marker ACA (anti-centromere antibody) and DNA for confocal microscopy. Compared to HCT116 p21+/+ cells, the congression defects in HCT116 p21−/− cells were enhanced, which were unchanged by the expression of wild type p21 or p21S130A, and marginally reduced by p21S130D (Figure 6B). In accordance with our previous data [6], the deficiency of p21 resulted in a high occurrence of chromosome segregation defects (Figure 6C and 6D, 2^nd^ panel, DNA staining), which were partially alleviated by wild type FLAG-p21 (Figure 6C and 6D, 3^rd^ panel). While p21S130A was able to mitigate these failures in a marginal extent, p21S130D expressing cells exhibited defects comparable to p21-deficient cells (Figure 6C and 6D, 4^th^ and 5^th^ panel), indicative of the importance of subsequent dephosphorylation of p21 during mitosis.

## DISCUSSION

In the present work we show that S130 of p21 is mainly phosphorylated by Cdk1/cyclin B1 during mitosis, which attenuates its stability and reduces its binding to Cdk1/cyclin B1. This regulation is of importance for a proper mitotic progression evidenced by extended prometaphase and metaphase in cells expressing the non-phosphorylatable mutant FLAG-p21S130A or the phosphomimetic FLAG-p21S130D, respectively. Interfering with this phosphorylation leads further to defective segregation and delayed mitotic exit indicating that a precise control of p21 is important for a successful mitotic progression.

Cdk1 becomes activated for the mitotic entry, its activity increases at prophase, reaches its peak shortly after nuclear envelope breakdown, remains almost constant in metaphase and is immediately reduced at the onset of anaphase by degradation of its regulatory subunit cyclin B1 [33–37]. It has been revealed that different thresholds of Cdk1/cyclin B1 trigger different events throughout mitosis by using FRET-biosensors [37, 38]. However, it is not totally understood how the activity of Cdk1/cyclin B1 is precisely regulated throughout mitosis. Based on our data, we suggest that, to reach its maximal activity necessary for mitotic events, Cdk1 is able to get rid of the inhibitory effect of p21 by phosphorylating its S130 residue to enhance its degradation and reduce its binding due to an eventual confirmation change. Indeed, the phosphorylation pattern of endogenous p21 reflects the Cdk1 activity during the mitotic progression. This observation is further underscored by showing that inhibition of Cdk1/cyclin B1, by either depletion of cyclin B1 or using the specific Cdk1 inhibitor RO-3306, reduces strongly the phosphorylation signal of p21. Moreover, we show that Cdk1/cyclin B1 phosphorylates S130 of p21 *in vitro* as well as *ex vivo*. In support of our notion, mass spectrometry analysis revealed that S130 in p21 is indeed phosphorylated in mitotic HeLa cells [39, 40], although this residue is also phosphorylated by Cdk2/cyclin E in the S phase [14], Cdk6/cyclin K upon viral infection [15] and ERK2 after mitogenic stimuli [24]. These data imply that S130 is an important residue of p21 to cope with its multiple functionalities upon diverse stimuli in different cell cycle stages. Interestingly, mitotic p21 is degraded by the APC/C(Cdc20)-mediated proteasome pathway [28]. In this context, we suggest that phosphorylation of S130 in p21 by Cdk1 could facilitate the access of APC/C(Cdc20) and promotes its degradation. In fact, both endogenous phosphorylated p21 as well as the Cdk1 phosphomimetic mutant p21S130D are less stable and degraded during mitosis.

We showed previously that p21 deficiency causes segregation defects which could be rescued by inhibiting Cdk1 activity with the Cdk1 inhibitor RO-3306 or by adding back wild type p21 [6]. Interfering with the phosphorylation at S130 leads also to defective segregation and prolonged mitotic duration suggesting that a precisely controlled functional p21 ensures an errorless progression through mitosis by fine-tuning Cdk1 activity. Cdk1/cyclin B1 controls various key steps in early mitosis like bipolar spindle assembly [41] and chromosome condensation [42] and its timely inactivation is essential for the initiation of anaphase and contributing to its irreversibility [43]. Defects in those events result in failed chromosome segregation in anaphase. Moreover, we have shown that loss of mitotic p21 reduces the activity of Aurora B and induces mislocalization of MKLP1 [6]. It is thus conceivable that phosphomimetic unstable p21S130D, similar to loss of p21, resulting in a hyperactive Cdk1, and non-phosphorylatable stable p21, attenuating the activity of Cdk1, could disrupt the spatially and timely regulated thresholds of Cdk1 activity and consequently its controlled mitotic events, leading further to segregation failures in anaphase. In particular, the phosphomimetic p21S130D induces more defective segregation associated with prolonged metaphase suggesting a timely dephosphorylation of p21 by phosphatases is required for the onset of anaphase and for ensuring the control over the remaining Cdk1 in late mitosis.

Taken together, we show here that p21 is phosphorylated at S130 by Cdk1/cyclin B1 during mitosis influencing its stability/degradation and ensuring a successful mitotic progression. Given that p53, the major transcriptional activator of p21, is the most frequently mutated gene in human cancer [44] and that hyperactive Cdk1 contributes to the development of different types of cancer [45], it is of clinical importance to study the function of p21 in cancer cells, its relevance in drug sensitivity and in tumor recurrence.

## MATERIALS AND METHODS

### Cell culture, inhibitors, transfections and time-lapse imaging

HCT116 p21+/+, HCT116 p21−/−, HeLa, MDA-MB-231, MCF7 and U2OS cells were cultured as instructed. Stable HeLa 776-6, expressing shRNA targeting cyclin B1, were generated as described [18, 19]. To synchronize cells in prometaphase, cells were treated with 50 ng/ml nocodazole (Sigma-Aldrich, Taufkirchen). Thymidine (Sigma-Aldrich) synchronization and release was performed as described [46]. The Plk1 inhibitor BI2536 (25 nM) was obtained from Selleck Chemicals LLC (Houston, USA), the specific Cdk1 inhibitor RO-3306 (9 μM) and the MAP cascade inhibitor PD98059 (10 μM) from Merck Millipore (Darmstadt). λ-Phosphatase (λ-PPase) was purchased from NEB (Frankfurt), MG132 (Z-Leu-Leu-al; 10 μM), cycloheximide (25 μg/ml) and DMSO from Sigma-Aldrich, the calpain inhibitor PD150606 (200 μM) from Santa Cruz (Heidelberg), and the pan-caspase inhibitor Z-VAD-FMK (Z-VAD; 20 μM) from Enzo Life Science GmbH (Lörrach). siRNA (10 to 20 nM) was transiently transfected with Oligofectamine™ (Life Technology). siRNAs targeting p21 (sense: ACACCUCCUCAUGUACAUAUU and antisense: AAUAUGUACAUGAGGAGGUGU), cyclin B1 (sense: GAAAUGUACCCUCCAGAAATT and antisense: GCUGACCCUGAAGUUCAUCUU) and Cdk2 (sense: ACACUCACCUUCUAGUCUUUU and antisense: AAGACUAGAAGGUGAGUGUUU) were manufactured by Sigma-Aldrich. Control siRNA was obtained from Qiagen (Hilden). For transient transfections with pBI-p21 and its constructs, electroporation was used (250 V, 250 μF, 500 Ω). The generation of the stable cell line HCT116 with H2B-tdTomato and the performance of time-lapse imaging are described [6]. FLAG constructs were transfected with FuGENE^®^ HD in a ratio 1:3 (Promega, Mannheim).

### Construction of DNA plasmids and recombinant protein expression

Full-length human p21 cDNA (pOTB7-vector) was obtained from ImaGenes GmbH (Berlin). By use of primers (5′-GATAGGATCCAGATGTCAGAACCGGCTGGGGATGTC-3′ and 5′-GACAGCGGCCGCTTAGGGCTTCCTCTTGGAGAAGATCAG-3′), the cDNA was amplified by PCR and subcloned into the *Bam*H I/*Not* I sites of the pGEX-5×3 vector (GE Healthcare, Munich), into the *Eco*R I/*Bam*H I sites (5′-AATTGAATTCTATGTCAGAACCGGCTGGGGATGTC-3′ and 5′-GATAGGATCCTTAGGGCTTCCTCTTGGAGAAGATCAG-3′) of the 3xFlag-CMV 7.1 vector (Sigma), and into the *Bam*H I/*Hin*d III sites of the pBI-CMV3 vector (Clontech), described in Kreis *et. al* [6]. Point mutations were generated with the QuikChange site-directed mutagenesis kit (Stratagene, Amsterdam) using the following primers: for T57A: 5′-GACTTTGTCACCGAGGCACCACTGGAGGGTG-3′, 5′-CACCCTCCAGTGGTGCCTCG-GTGACAAAGTC-3′; for S98A: 5′- CGGCC TGGCACCGCACCTGCTCTGC-3′, 5′- GCAGAGCAG GTGCGGTGCCAGGCCG-3′; for S130A: 5′-CAGG CTGAAGGG-GCCCCAGGTGGAC-3′, 5′- GTCCACC TGGGGCCCCTTCAGCCTG-3′; and for S130D: 5′- AGCAGGCTGAAGGGGACCCAGGTGGACCTG-3′, 5′-CAGGTCCACCTGGGTCCC-CTTCAGCCTGCT-3′. All mutant constructs were confirmed by DNA sequencing. GST-tagged MCAK was cloned as described [22]. Recombinant GST-p21 and its mutants were induced and expressed in Escherichia coli BL21(DE3)-CodonPlus cells and purified using Glutathione Sepharose™ 4B beads (GE Healthcare).

### Western blot analysis, FACS, pulldown, immunoprecipitation and kinase assays

Cells were lysed in RIPA buffer and Western blot analysis was performed, as described [46]. Cytoplasmic and nuclear fractionation was performed as instructed (Active Motif, La Hulpe, Belgium). Cell cycle was analyzed using a FACSCalibur™ (BD Biosciences, Heidelberg), as described [17]. Pulldown assay was performed with GST, wild type GST-p21, GST-p21S130A and GST-p21S130D, cell extracts of nocodazole synchronized HCT116 p21−/− cells and Glutathione Sepharose™ beads (GE Healthcare) in binding puffer containing 10% glycerol and 0.1% NP-40 in PBS. Immunoprecipitation was carried out as described [6]. Following antibodies were used: mouse monoclonal antibodies against cyclin B1 (GNS1), GST (B-14), Plk1 (F-8), p53 (DO-1), Cdk1 (Cdc2 p34), and rabbit polyclonal antibodies against cyclin B1 (H-433) and Cdk2 (M2; Santa Cruz, Heidelberg); mouse or rabbit monoclonal antibodies against p21 (DCS60 or 12D1; Cell Signaling, Beverly), rabbit polyclonal anti-phospho-histone H3 (S10) (p-HH3; Merck Millipore) and mouse monoclonal antibodies against β-actin and FLAG^®^ M2 (Sigma-Aldrich, Taufkirchen). Mouse monoclonal antibody against calnexin was from BD Biosciences and against lamin B1 from MBL (Woburn, USA). To generate a polyclonal antibody against p21 phosphorylated on S130, rabbits were immunized using the peptide GEQAEGpSPGGPGD (p = phospho), and antibodies were affinity purified (Eurogentec, Seraing, Belgium). Kinase assay *in vitro* was performed as described [22]. Cdk1/cyclin B1 kinase was purchased from NEB. Kinase assay *ex vivo* was performed with HeLa cells transfected with FLAG-p21 or FLAG-p21S130A. Cells were synchronized with nocodazole and immunoprecipitated with FLAG^®^ M2 beads (Sigma). The beads were subjected with radioactive ATP (Perkin Elmer, Hamburg) without adding additional kinase and incubated for 30 min at 37°C with or without inhibitors.

### Indirect immunofluorescence microscopy and confocal laser scanning microscopy

For indirect immunofluorescence staining, cells were seeded on Nunc™ Lab-Tek™ II CC2™ chamber slides from Thermo Fisher Scientific (Schwerte). Briefly, cells were fixed for 15 min with 4% PFA and permeabilized for 5 min with 0.1% Triton™ X-100 at room temperature. The following primary antibodies were used for staining: polyclonal rabbit antibody against pericentrin (abcam^®^, Cambridge, UK), monoclonal mouse antibody against FITC-conjugated α-tubulin (Sigma-Aldrich) and human immune serum against centromere (anti-centromere antibody, ACA, ImmunoVision, Springdale, USA). Cy3 and Cy5-conjugated secondary antibodies were obtained from Jackson Immunoresearch (Newmarket, UK). DNA was stained using DAPI (4′,6-diamidino-2-phenylindole-dihydrochloride, Roche). Slides were examined using an AxioObserver.Z1 microscope with a HCX PL APO CS 63.0×1.4 oil UV objective (Zeiss, Göttingen) and images were taken using a confocal laser scanning microscope (CLSM, Leica CTR 6500, Heidelberg).

### Statistical analysis

Student's *t*-test (two tailed and paired or homoscedastic) was used to evaluate the significance of difference between the different conditions. Difference was considered as statistically significant when p < 0.05.

## Abbreviations

Cdk1: cyclin-dependent kinase 1
Cip1: Cdk-interacting protein 1
MCAK: mitotic centromere-associated kinesin
CHX: cycloheximide
